# The one, the few and the many

**DOI:** 10.1192/bjb.2020.82

**Published:** 2020-10

**Authors:** Norman Poole

**Affiliations:** 1 South West London and St George's Mental Health NHS Trust

**Keywords:** Inequality, poverty, service users

## Abstract

Inequality is a significant and reversible risk factor for mental disorders which demonstrates the essentially political nature of psychiatry. The *BJPsych Bulletin* is pleased to make space for those at the frontline of this inequality. Two experts by experience explain how financial concerns exacerbated their distress and the benefit of social interventions.

Psychiatry's great appeal is also its Achilles’ heel. It embraces a multitude of perspectives which can appear mutually incompatible, drawing accusations of vagueness and incoherence. This themed edition of the *BJPsych Bulletin* will, I hope, demonstrate that such criticisms are wrongheaded. It has been a pleasure to work with Peter Byrne to produce this edition on inequality, a recognised but still under-appreciated driver of mental illness about which he is passionate and persuasive. Although epidemiological studies can feel rather detached or aloof, they have identified some of the most significant and well-replicated risks for developing mental disorder, something that cannot be said for the majority of experiments in neuroscience, despite its glossy allure. Nevertheless, accommodating psychiatry's various perspectives involves somehow tethering known risk factors to plausible biological mechanisms, whether one considers the suffering a disorder or not. For clinicians, it is the individual patient who must be the focus, but too often our concern with odds ratios and objective data can come across as alienating. These different and incommensurate levels of analysis remind me of the various forms of government – by the one, the few or the many – and that psychiatry is inextricably political. Inequality is a reversible cause of mental disorder, and inequality is a political choice. If doctors find it hard to be heard in the political realm, how much harder must it be for those who suffer the consequences of inequality? I am especially pleased to include in this article contributions from two experts by experience, Nikita Egan and Ben Russell, both from Equally Well UK (https://equallywell.co.uk/), a collaborative – led by Centre for Mental Health in partnership with Rethink Mental Illness – that champions parity of esteem. Their moving accounts of illness and alienation underscore why we do what we do and, I hope, establish the Bulletin's increased focus on co-production and, in our own small way, sharing the levers of power. Over to you, Nikita and Ben.

## Nikita Egan, Expert by Experience, Equally Well UK

‘We as a species are not very good at asking for help. In doing so, we are admitting, to ourselves at least, that we aren't as independent as we might have hoped – that we're fallible. If we consider this natural discomfort felt when asking for help, is it any wonder that applying for benefits such as UC [universal credit] and PIP [personal independence payment] can often seem like a humiliating ordeal?

‘It was a previous CPN [community psychiatric nurse] who advised me to apply for PIP, stating that I shouldn't see it as a benefit, but more to put me on an equal playing field with my peers who didn't face a daily struggle with their mental health. When the form did arrive it seemed condescending, but also forced me to look inwards to my own daily difficulties. When the form was complete, I asked my CPN to read it. Did she think I'd exaggerated? Did she not really think I should apply? Thoughts like this were a daily monologue in my head, but having read horror stories from other applicants, I didn't hold out much hope anyway. Being awarded the higher rate for daily living and lower for mobility was such a contradictory feeling, especially as I didn't have a face-to-face assessment. Is this my life now, living on benefits? Accepting handouts?

‘The time to apply for UC didn't arrive until 2018 when my marriage ended. I had nothing except my PIP and no choice but to apply online for UC. In my head all I could think of was ‘going on the dole’. As I filled in my disability, details of the emotions of the PIP application process came flooding back.

‘The face-to-face assessment was terrifying, especially finding out that my date and time were the same as the letter given to 12 other people. The paranoia was terrible, and so many obscure ideas ran through my mind. I began to panic as I scanned the room; everyone else seemed to have a physical impairment. As it turned out the assessment was with a compassionate nurse. My form and records had been checked; I just had to confirm some details and give a copy of my prescription.

‘PIP and UC – based on the decision that I have limited capacity to undertake paid work – allow me to pay my rent and bills, and also to enjoy an albeit thrifty life with my children. Many others are not as lucky, and don't have the support to make and endure application processes.

‘Personally, I don't think of myself as living on benefits. I think of myself as living, however I can.’

## Ben Russell, Expert by Experience, Equally Well UK

‘It's difficult to focus cause I'm being harangued from all sides. I run from room to room to escape, but they follow me. I turn around suddenly, hoping to catch them in the act, but there's no one else there. I am alone. I realise, all of a sudden, that I'm ill again. I hear a hissing sound and spin around and see a fox standing there with blood-red eyes and its hackles raised. I scream and *run* to the next room, but the fox is already in there. In fact, it's in every room I run to waiting for me. It's difficult because knowing I'm ill doesn't stop me being ill. I go into town hoping that the fresh air will do me good, but there are people following me. If they catch me, they'll do experiments on me, so I run back to my flat and lock the door behind me. I don't know how long I've been ill for, but there's a mound of envelopes in my letterbox. More than likely bills. But I can't function, let alone pay them. I also don't remember the last time I ate. Nothing makes sense.

‘I've also not been paying my bills. The mortgage company send me threatening letters, as do the council. I'm also unable to work. My phone is vibrating on the coffee table. There're seven missed calls. I answer warily, ready to be berated some more. It's my nurse. She says that she's been trying to contact me for days. I tell her that I'm not right in the head. An indeterminable amount of time passes before my nurse comes to my flat. She says that I need to be in hospital, but I'm worried about my flat. I get personal independence payments, but they're not enough to cover my mortgage. I was working as a hod carrier, but then I became unwell with my mental health and no longer have a job. My nurse is insistent anyway. I go to hospital. On the ward, my family are contacted. They arrange to help pay my bills, which is the luckiest thing, because I was about to lose my flat. The important thing, they said, was getting well. And so many people in my position don't have that chance. The crisis team also help me get in touch with my creditors and arrange payment plans.’

## About the author

**Norman A. Poole** is Editor of the *BJPsych Bulletin* and a consultant neuropsychiatrist at St George's Hospital, South West London and St George's Mental Health NHS Trust, UK.

